# Large Neutral Amino Acid Supplementation Exerts Its Effect through Three Synergistic Mechanisms: Proof of Principle in Phenylketonuria Mice

**DOI:** 10.1371/journal.pone.0143833

**Published:** 2015-12-01

**Authors:** Danique van Vliet, Vibeke M. Bruinenberg, Priscila N. Mazzola, Martijn H. J. R. van Faassen, Pim de Blaauw, Ido P. Kema, M. Rebecca Heiner-Fokkema, Rogier D. van Anholt, Eddy A. van der Zee, Francjan J. van Spronsen

**Affiliations:** 1 University of Groningen, University Medical Center Groningen, Beatrix Children’s Hospital, Groningen, The Netherlands; 2 University of Groningen, Center of Behavior and Neurosciences, Department of Molecular Neurobiology, Groningen, The Netherlands; 3 University of Groningen, University Medical Center Groningen, Department of Laboratory Medicine, Groningen, The Netherlands; 4 Independent Researcher, Deventer, The Netherlands; University of Arkansas for Medical Sciences; College of Pharmacy, UNITED STATES

## Abstract

**Background:**

Phenylketonuria (PKU) was the first disorder in which severe neurocognitive dysfunction could be prevented by dietary treatment. However, despite this effect, neuropsychological outcome in PKU still remains suboptimal and the phenylalanine-restricted diet is very demanding. To improve neuropsychological outcome and relieve the dietary restrictions for PKU patients, supplementation of large neutral amino acids (LNAA) is suggested as alternative treatment strategy that might correct all brain biochemical disturbances caused by high blood phenylalanine, and thereby improve neurocognitive functioning.

**Objective:**

As a proof-of-principle, this study aimed to investigate all hypothesized biochemical treatment objectives of LNAA supplementation (normalizing brain phenylalanine, non-phenylalanine LNAA, and monoaminergic neurotransmitter concentrations) in PKU mice.

**Methods:**

C57Bl/6 *Pah-enu2* (PKU) mice and wild-type mice received a LNAA supplemented diet, an isonitrogenic/isocaloric high-protein control diet, or normal chow. After six weeks of dietary treatment, blood and brain amino acid and monoaminergic neurotransmitter concentrations were assessed.

**Results:**

In PKU mice, the investigated LNAA supplementation regimen significantly reduced blood and brain phenylalanine concentrations by 33% and 26%, respectively, compared to normal chow (*p*<0.01), while alleviating brain deficiencies of some but not all supplemented LNAA. Moreover, LNAA supplementation in PKU mice significantly increased brain serotonin and norepinephrine concentrations from 35% to 71% and from 57% to 86% of wild-type concentrations (*p*<0.01), respectively, but not brain dopamine concentrations (*p* = 0.307).

**Conclusions:**

This study shows that LNAA supplementation without dietary phenylalanine restriction in PKU mice improves brain biochemistry through all three hypothesized biochemical mechanisms. Thereby, these data provide proof-of-concept for LNAA supplementation as a valuable alternative dietary treatment strategy in PKU. Based on these results, LNAA treatment should be further optimized for clinical application with regard to the composition and dose of the LNAA supplement, taking into account all three working mechanisms of LNAA treatment.

## Introduction

Phenylketonuria (PKU; OMIM 261600) is the first disorder in which severe neurocognitive dysfunction could be prevented by dietary treatment. It is caused by a deficient activity of the hepatic enzyme phenylalanine hydroxylase (PAH; EC 1.14.16.1), normally converting phenylalanine (Phe) to tyrosine. If left untreated, classical PKU symptomatology is almost exclusively restricted to the brain, including severe neurocognitive dysfunction, seizures, and psychiatric problems, correlating with high blood Phe concentrations [1].

This selective brain vulnerability to high blood Phe concentrations is hypothesized to be related to the transport characteristics for Phe at the blood-brain barrier (BBB) [2,3]. At the BBB, the large neutral amino acid transporter 1 (LAT1) is the predominant transport system for all large neutral amino acids (LNAA), and is saturated for >95% [4]. Combined with the fact that LAT1 shows a high affinity to Phe, increased blood Phe concentrations strongly increase brain Phe influx, outcompeting the transport of other LNAA [5–8]. Based on both the increased Phe and the decreased non-Phe LNAA transport across the BBB, different brain biochemical disturbances underlie brain dysfunction in PKU [3,9]. High brain Phe concentrations have been found to be neurotoxic and to affect brain metabolism [10–14], while reduced brain availability of non-Phe LNAA has been related to impaired cerebral protein synthesis [6,15]. In addition, impaired cerebral monoaminergic neurotransmitter synthesis may result from outcompeted brain uptake of their amino acid precursors tyrosine and tryptophan [16], and/or from an inhibitory effect of high brain Phe concentrations on tyrosine hydroxylase (TH) and tryptophan hydroxylase (TPH) [17].

Thus far, blood Phe reduction has been the primary target of treatment in PKU. This can be accomplished by a severe Phe-restricted diet and, in some patients, by pharmacological treatment with tetrahydrobiopterin. However, early- and continuously treated PKU patients still show impaired executive functioning and are prone to develop anxiety and depressive symptoms [18,19]. Moreover, the Phe-restricted diet is socially demanding and hard to comply with [20]. Therefore, an alternative pathophysiology-based treatment strategy directly targeting the brain is required. One such possible treatment strategy includes supplementation of LNAA (without Phe) that aims to restore the disturbed LNAA transport across the BBB without dietary Phe restriction. Based on aforementioned hypotheses on PKU pathophysiology, LNAA supplementation could serve to: 1) decrease brain Phe, 2) increase brain non-Phe LNAA, and/or 3) increase brain monoaminergic neurotransmitter concentrations [21].

Previous research on LNAA treatment in PKU has primarily focused on brain and blood Phe reduction as treatment objective [22–28]. In addition, LNAA supplementation in PKU patients was recently found to increase blood and urine melatonin concentrations, which might reflect increased brain serotonin synthesis [29]. However, not all brain biochemical treatment objectives have been investigated. In consequence, optimal composition and dosing of LNAA treatment is currently unknown, limiting its clinical application. As a first step to develop an optimal LNAA treatment regimen, the aim of the present study was to assess all abovementioned hypothesized biochemical treatment objectives of LNAA supplementation in a PKU mouse model.

## Material & Methods

### Animals

To establish a new breeding colony, breeding pairs of C57Bl/6 *Pah-enu2* mice had been kindly provided by Prof. B. Thöny from the division of Clinical Chemistry and Biochemistry of the University Children’s Hospital in Zurich in Switzerland. From heterozygous (+/-) mating, wild-type (WT, +/+), heterozygous, and PKU (-/-) mice of both sexes were obtained. After weaning at four weeks of age, genetic characterization was performed by quantitative PCR analysis on DNA extracted from ear tissue. Animals were housed individually at 21±1°C on a 12-hr light-dark cycle (7:00 am-7:00 pm), and water and AM-II food pellets (Arie Block BV, Woerden, The Netherlands) were offered *ad libitum*. In total, 42 WT (21 male, 21 female) and 46 PKU (23 male, 23 female) mice were used. This study was carried out in strict accordance with the recommendations in the Guide for the Care and Use of Laboratory Animals of the National Institutes of Health (S1 ARRIVE Checklist). The protocol was approved by the Institutional Animal Care and Use Committee of the University of Groningen (Permit Number: 6504A).

### Experimental design

At postnatal day 37, animals were assigned to one of three treatment groups based on genotype and sex, receiving either normal chow, a high-protein diet, or a LNAA supplemented diet. Dietary treatment was continued for six weeks. During the first week of treatment, body weight and food intake were measured daily, after which body weight and food intake were determined at weekly intervals. Food intake was manually assessed by the difference between food given and left on the cages’ tops using a scale. Spilled food was not measured because it has been shown to represent less than 0.1 g/day/mouse [30]. After six weeks of dietary treatment, animals were euthanized by combined heart puncture and decapitation under inhalation-anesthetics with isoflurane 2–3 hours after the beginning of the light phase.

### Experimental diets

The basal diet during the experiment was based on the composition of AIN-93M [31], which was also administered in unadjusted form to the untreated control group (normal chow). The experimental LNAA diet was based on the LNAA regimen as used in the study by Pietz et al. [26]. It was produced by adding LNAA to the basal diet at the expense of cornstarch on a weight-for-weight basis. The added amount of LNAA was equal to the amount of (natural) protein in the basal diet, in effect doubling the amount of protein/amino acids in the LNAA supplemented diet. The LNAA added in the LNAA supplemented diet included equal amounts of l-tyrosine, l-tryptophan, l-valine, l-isoleucine, l-leucine, l-methionine, and l-histidine. To control for the extra amount of protein in the LNAA supplemented diet, a high-protein control diet was included. This high-protein diet was produced by adding extra casein to the AIN-93M diet at the expense of cornstarch on a weight-for-weight basis, and was calculated to result in an isonitrogenic and isocaloric control diet for the experimental LNAA diet. Diets were prepared by Research Diet Services B.V. (Wijk bij Duurstede, The Netherlands).

### Biochemical analyses

To obtain plasma and brain material for biochemical analyses, blood was collected by heart puncture and whole brains were removed. Blood was centrifuged at 1500 g for 10 min and plasma was collected and stored at -80°C until further analysis. The cerebrum was snap frozen in liquid nitrogen and stored at -80°C until further preparation. Frozen cerebrum was crushed in liquid nitrogen and divided into aliquots. Frozen brain powder for amino acid measurements was processed to 20% (weight: volume (w:v)) homogenates in phosphate-buffered saline (pH 7.4), and for tryptophan, indole and catecholamine measurements to 2% (w:v) homogenates in acetic acid (0.08 M). Brain homogenates were sonified on ice at 11–12 W. Next, samples were centrifuged at 800 rcf for 10 min (4°C), and the supernatant/internatant was put on ice to be used for further analysis.

For brain amino acid (except for tryptophan) measurements, norleucine in sulfosalicylic acid was added as an internal standard to the 20% brain homogenate (1:1, v:v). Samples were vortexed and centrifuged at 20.800 rcf for 4 min. Plasma amino acid measurements were performed according to the same method, using 50 μl plasma instead of 20% brain homogenate. Amino acid concentrations were measured with a method based on ion exchange chromatography with post column derivatization with Ninhydrin on a Biochrom 30+ analyser (Pharmacia Biotech, Cambridge, UK).

For tryptophan and monoaminergic neurotransmitter measurements, an anti-oxidative solution was prepared in demineralised water (0.4 g/l ascorbic acid and 1.616 g/l ethylenediaminetetraacetic acid). For tryptophan and indole measurements, 25 μl of the anti-oxidative solution was added to 25 μl of the 2% brain homogenate. For catecholamine measurements, 40 μl of the anti-oxidative solution was added to 10 μl of the 2% brain homogenate. Plasma tryptophan measurements were performed using 25 μl plasma instead of 2% brain homogenate. Analysis of tryptophan and monoaminergic neurotransmitter concentrations was performed using liquid chromatography in combination with isotope dilution mass spectrometry, essentially as described by Van de Merbel et al. [32].

### Statistical analyses

Statistical analyses were performed using the software IBM SPSS Statistics for Windows, Version 22.0. (Armonk, NY: IBM Corp.). Data of the one animal that was euthanized preterm, were excluded from analyses. All tests were performed two-sided at a significance level of α = 0.05.

Analyses on brain and blood biochemistry as well as on weekly food intake (per g body weight) were performed by two-way ANOVA with genotype and diet as independent variables. In case of not normally distributed data (assessed by Shapiro-Wilk test) or unequal variances (assessed by Levene’s test), analyses were performed on log-transformed data. If the interaction between genotype and diet and/or a main effect of diet was found to be significant, data were further analyzed by one-way ANOVA and Tukey’s *post-hoc* tests for PKU and WT mice separately.

The effect of dietary treatment on body weight was analyzed for WT and PKU male and female mice separately by repeated-measures ANOVA with one between factor (diet, three levels: normal chow, high-protein diet, and LNAA diet) and one within factor (time, 7 levels: 0, 1, 2, 3, 4, 5, and 6 weeks) and Tukey’s *post-hoc* analysis.

To investigate whether brain Phe concentrations in PKU mice were primarily determined by blood Phe concentrations or by dietary treatment, multiple linear regression analysis was performed with blood Phe concentrations and dietary treatment as independent variables.

Data are expressed as mean ± standard deviation (SD).

## Results

### Food and LNAA intake

Amino acid contents of the different diets are presented in Table 1.

**Table 1:** Nutritional content of the experimental diets (g/kg diet).

Nutritional content*		normal chow	LNAA diet		high-protein diet	
**Carbohydrates**		674	550		551	
**Fat**		41	41		41	
**Dietary fibre**		50	50		50	
**Protein**		124	248		248	
Amino acids**						
**LNAA**	L-Phenylalanine	6.0	6.1	(0.1)	12.2	(6.2)
	L-Tyrosine	4.8	20.1	(15.3)	12.1	(7.3)
	L-Valine	7.4	25.0	(17.6)	15.9	(8.5)
	L-Isoleucine	5.9	22.9	(17.0)	12.4	(6.4)
	L-Leucine	10.9	28.0	(17.1)	23.1	(12.2)
	L-Methionine	3.0	19.7	(16.7)	6.9	(3.9)
	L-Histidine	3.2	19.0	(15.8)	6.8	(3.5)
	L-Threonine	5.3	5.7	(0.3)	11.3	(5.9)
**non-LNAA**	L-Aspartic acid	9.3	9.7	(0.4)	19.4	(10.1)
	L-Serine	7.7	7.9	(0.2)	15.8	(8.1)
	L-Glutamic acid	28.2	29.9	(1.7)	59.6	(31.4)
	Glycine	2.7	2.7	(0.0)	5.2	(2.5)
	L-Alanine	3.9	4.2	(0.3)	8.2	(4.3)
	L-Lysine	9.1	9.9	(0.8)	19.5	(10.4)
	L-Arginine	4.2	4.7	(0.5)	9.5	(5.3)

Contents are not shown for L-Tryptophan, L-Proline, and L-cyst(e)ine, as these were not measured due to technical limitations. Differences with LNAA contents of normal chow are stated in brackets. LNAA, large neutral amino acid.

*Mineral and vitamin premixes were also included in accordance with the composition of the AIN93M diet [30].

**Dietary contents as measured in samples of prepared food pellets.

**Fig 1:**
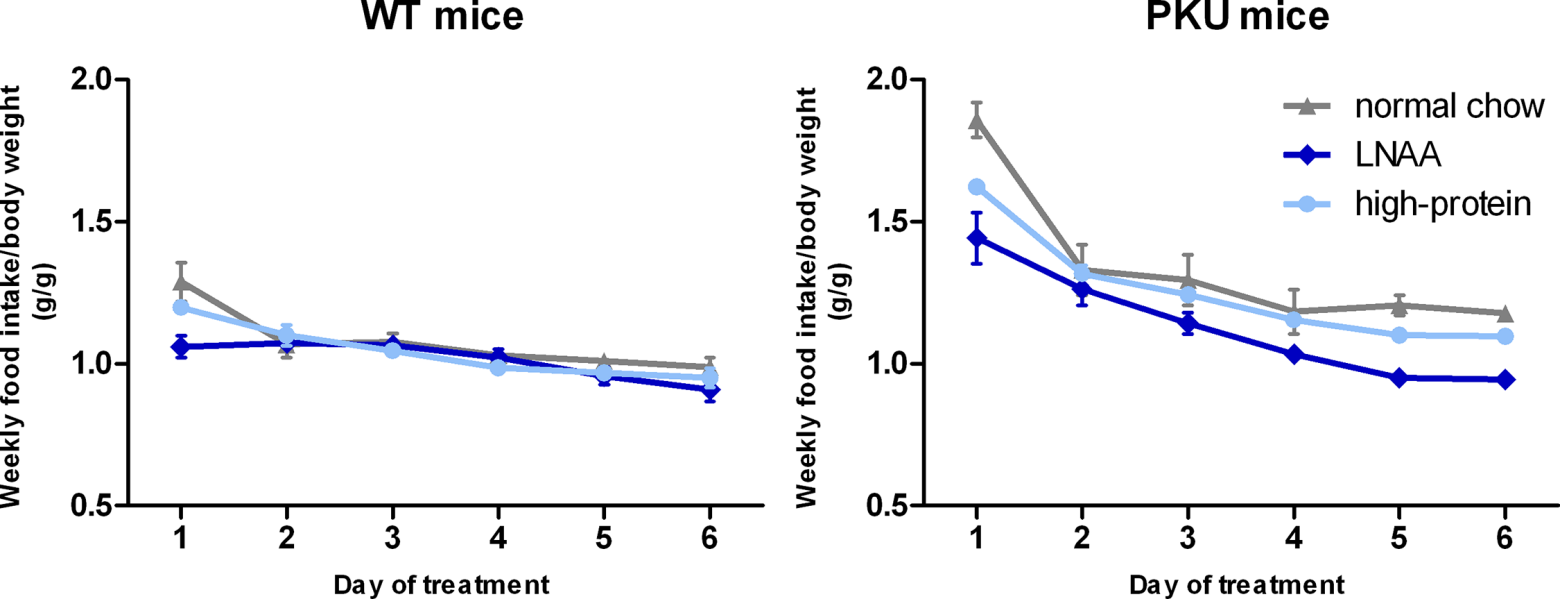
Weekly food intake for WT (A) and PKU (B) mice on different diets. Numbers of mice on normal chow, LNAA supplemented diet, and high-protein diet were n = 14, n = 14, and n = 14 for WT mice respectively, while being n = 15, n = 14, and n = 15 for PKU mice. Error bars represent SEM.

Weekly food intake (expressed as g food/g body weight/week), as shown in Fig 1, decreased during the first weeks for all treatment groups, stabilizing in the later weeks of dietary treatment. In both the fifth and sixth week of dietary treatment, two-way ANOVA analyses showed a significant main effect of genotype on food intake (*p*<0.01). Moreover, in both weeks, a significant main effect of dietary treatment (*p*<0.01), and a significant interaction between genotype and dietary treatment on food intake was observed (*p*<0.01 for week 5, *p*<0.05 for week 6). In PKU mice, food intake in both weeks was lower on high-protein diet than on normal chow (*p*<0.05), while being even lower on LNAA supplemented diet (*p*<0.01). In WT mice, food intake in both weeks did not significantly differ for any of the dietary treatments (*p* = 0.376 and *p* = 0.322). Based on the weekly food intakes and amino acid contents of the different diets, mean daily intakes of individual LNAA during the six week dietary treatment were calculated for all experimental groups as shown in the S1 Table.

### Body weight and general health

**Fig 2:**
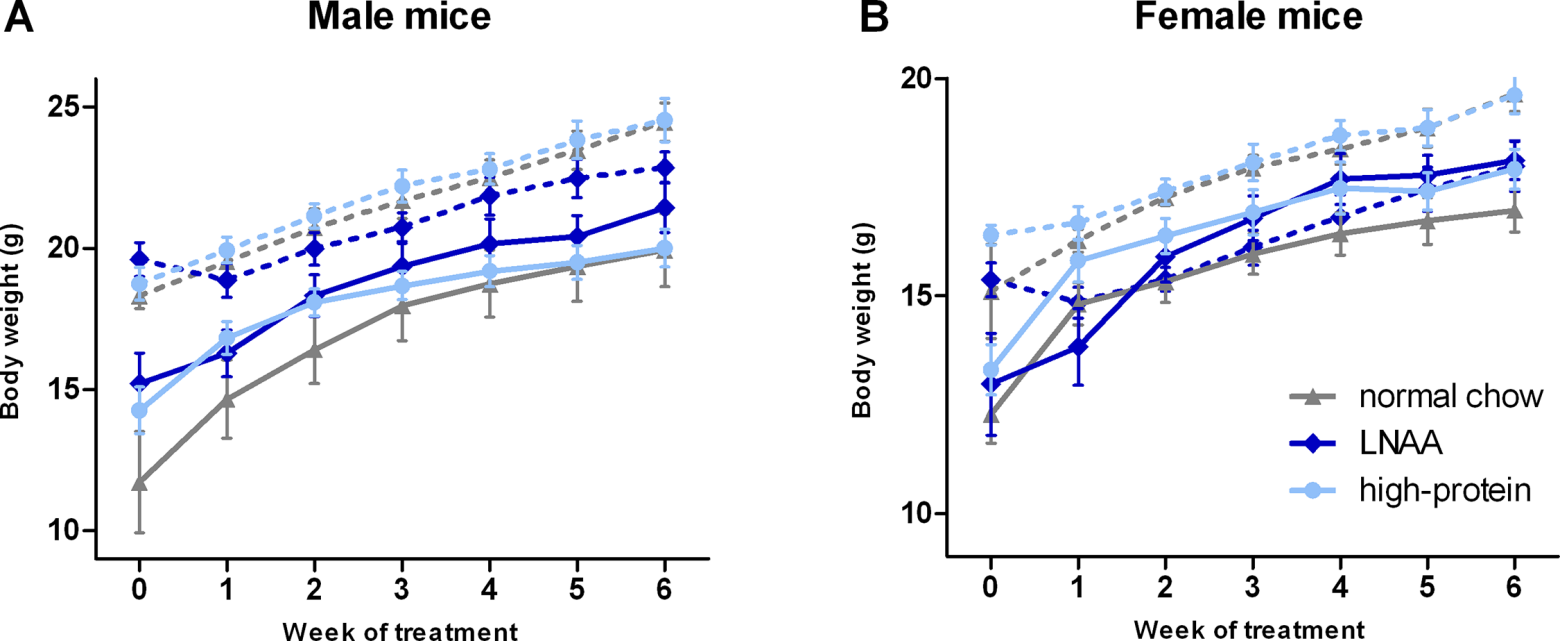
Body weights during the experiment. Mean body weights for A) male and B) female WT (dashed lines) and PKU (solid lines) mice on different diets. Numbers of mice on normal chow, LNAA supplemented diet, and high-protein diet were n = 14, n = 14, and n = 14 for WT mice respectively, while being n = 15, n = 14, and n = 15 for PKU mice. Error bars represent SEM.

At initiation of treatment, body weight of PKU mice (male 13.7 ± 3.6 g; female: 12.8 ± 2.1 g) was lower than of WT mice (male 18.9 ± 1.5 g; female: 15.6 ± 1.8 g), but did not significantly differ between dietary groups in PKU nor WT mice. Body weight curves during treatment were significantly lower for WT female mice on LNAA supplemented diet compared to either normal chow (*p*<0.05) or high-protein diet (*p*<0.01), but did not significantly differ between dietary treatments for WT male (*p* = 0.485) and PKU male or PKU female mice (*p* = 0.397 and *p* = 0.343) (Fig 2).

During the experiment, one animal (WT female on LNAA supplemented diet) was euthanized on the 19^th^ day after inclusion, because of too much weight loss. Pathological examination showed hydrocephalus, which is sometimes found in C57Bl/6 (both WT and PKU) mice.

### Plasma amino acids

**Fig 3:**
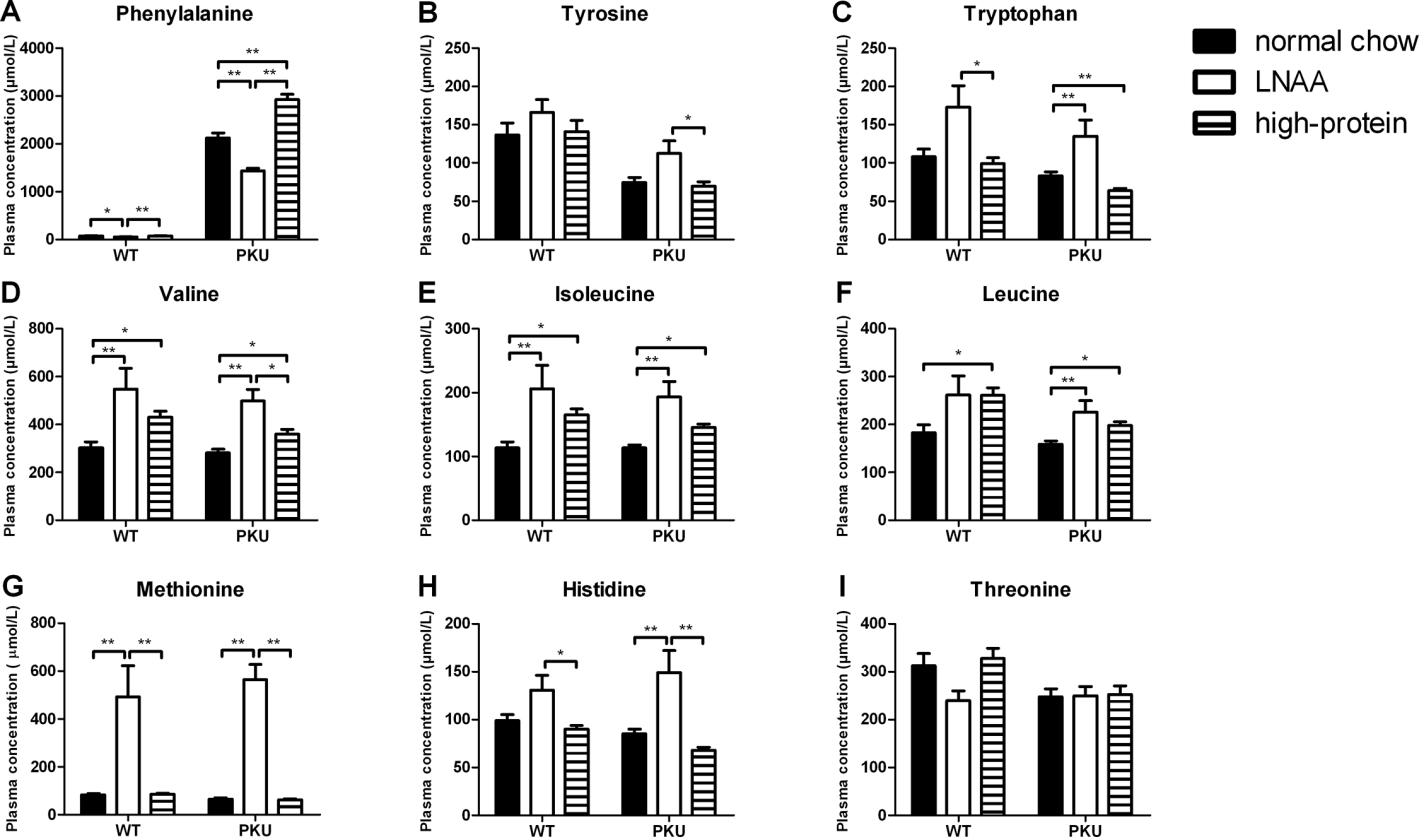
Plasma LNAA concentrations. Plasma concentrations of A) phenylalanine, B) tyrosine, C) tryptophan, D) valine, E) isoleucine, F) leucine, G) methionine, H) histidine, and I) threonine in WT and PKU mice after six weeks of receiving different diets. Numbers of mice on normal chow, LNAA supplemented diet, and high-protein diet were n = 14, n = 12, and n = 14 for WT mice respectively, while being n = 14, n = 12, and n = 15 for PKU mice. Untransformed data are expressed as mean ± SEM. * *p*<0.05; ** *p*<0.01; § *p*<0.05 and §§ *p*<0.01 compared to WT mice on normal chow.

Plasma concentrations of individual LNAA in PKU and WT mice on control and LNAA supplemented diets are depicted in Fig 3. Two-way ANOVA analyses showed a significant main effect of genotype on plasma Phe, tyrosine, tryptophan (*p*<0.01), and threonine concentrations (*p*<0.05). In PKU compared to WT mice on normal chow, plasma Phe concentrations were increased by almost 30-fold. Plasma tyrosine, tryptophan, and threonine concentrations in PKU mice were reduced to 55%, 77%, and 79%, respectively, of concentrations in WT mice on normal chow. For other LNAA, no significant main effect of genotype was observed on plasma concentrations.

A significant main effect of dietary treatment was observed on plasma concentrations of all LNAA except for threonine (*p* = 0.073 for threonine; *p*<0.05 for tyrosine; *p*<0.01 for all other LNAA). Moreover, two-way ANOVA analyses showed a significant interaction between genotype and dietary treatment on plasma Phe and methionine concentrations (*p*<0.01 for both). In PKU mice, plasma Phe concentrations on LNAA supplemented diet were significantly reduced to 67% of concentrations on normal chow (*p*<0.01), while plasma Phe concentrations on high-protein diet were significantly higher than on normal chow (*p*<0.01). In WT mice on LNAA, plasma Phe concentrations were reduced compared to control diets (p<0.05), but concentrations on high-protein diet did not significantly differ from those on normal chow (*p* = 0.929). Plasma concentrations of supplemented LNAA were higher on LNAA supplementation compared to control diets, both in PKU and WT mice, although this did not reach statistical significance for all LNAA.

Plasma concentrations of non-LNAA amino acids in PKU and WT mice on control and LNAA supplemented diets are presented in S2 Table. In both PKU and WT mice, plasma glycine and lysine concentrations on LNAA supplemented diet were lower compared to control diets (*p*<0.05), just not reaching statistical significance for lysine in PKU mice (*p* = 0.051). In addition, plasma serine concentrations were lower on LNAA supplementation compared to control diets in WT mice (*p*<0.05), but not in PKU mice (*p* = 0.407).

### Brain amino acids

**Fig 4:**
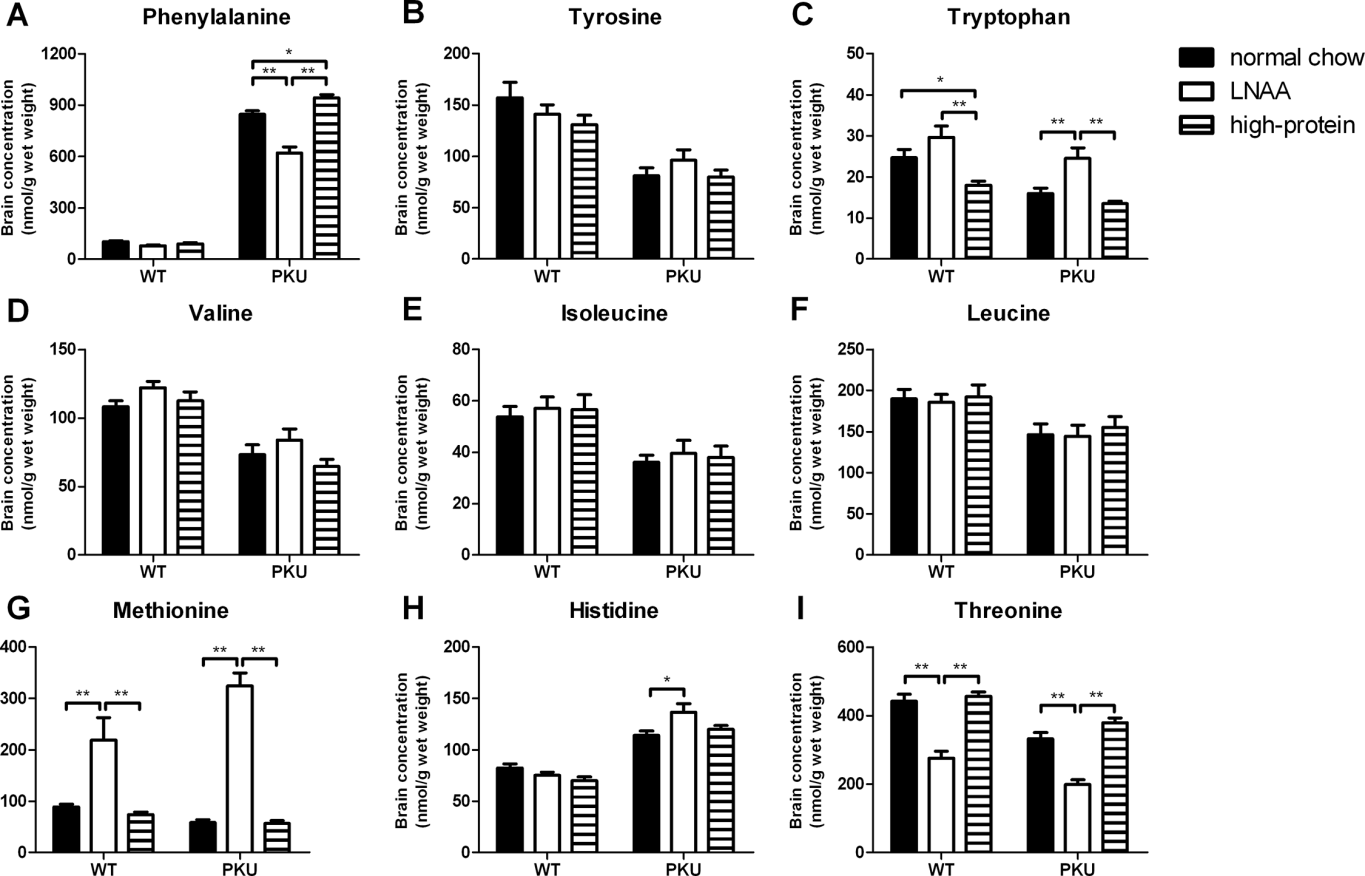
Brain LNAA concentrations. Brain concentrations of A) phenylalanine, B) tyrosine, C) tryptophan, D) valine, E) isoleucine, F) leucine, G) methionine, H) histidine, and I) threonine in WT and PKU mice after six weeks of receiving different diets. Numbers of mice on normal chow, LNAA supplemented diet, and high-protein diet were n = 13, n = 12, and n = 14 for WT mice respectively, while being n = 14, n = 12, and n = 14 for PKU mice. Untransformed data are expressed as mean ± SEM. * *p*<0.05; ** *p*<0.01; § *p*<0.05 and §§ *p*<0.01 compared to WT mice on normal chow.

Brain concentrations of individual LNAA in PKU and WT mice on control and LNAA supplemented diets are depicted in Fig 4. Two-way ANOVA analyses showed a significant main effect of genotype on brain concentrations of all LNAA except for methionine (*p*<0.01 for all LNAA but methionine, *p* = 0.523 for methionine). In PKU compared to WT mice on normal chow, brain Phe concentrations were increased by 8.3-fold. Also, brain histidine concentrations in PKU mice were elevated to 139% of WT concentrations on normal chow. Brain concentrations of all other non-Phe LNAA but methionine were reduced in PKU mice on normal chow, ranging from 52% of concentrations in corresponding WT mice for tyrosine to 77% for leucine.

A significant main effect of dietary treatment was observed on brain Phe, tryptophan, methionine, and threonine concentrations (*p*<0.01 for all). Moreover, two-way ANOVA analyses showed a significant interaction between genotype and dietary treatment on brain Phe, methionine, and histidine concentrations (*p*<0.01 for Phe and methionine, *p*<0.05 for histidine). Brain Phe concentrations in PKU mice on LNAA supplementation were reduced to 74% of concentrations on normal chow (*p*<0.01), still being 6.1-fold higher when compared to WT concentrations on normal chow. Brain Phe concentrations in PKU mice on high-protein diet were higher than in PKU mice on normal chow (*p*<0.05). Brain tryptophan, histidine, and methionine concentrations in PKU mice on LNAA supplementation were significantly higher when compared to PKU mice on normal chow (*p*<0.05), resulting in concentrations of 100% and 166% of WT concentrations on normal chow for tryptophan and histidine, and an elevation by 3.6-fold for methionine. In contrast, brain threonine concentrations in PKU mice on LNAA supplementation were lower when compared to PKU mice on control diets (*p*<0.01).

In WT mice, brain Phe concentrations on LNAA supplementation tended to be lower when compared to normal chow, although this just did not reach statistical significance (*p* = 0.053). Similar to PKU mice, brain methionine concentrations were higher, whereas brain threonine concentrations were lower on LNAA supplementation as compared to control diets (*p*<0.01 for all).

Brain concentrations of non-LNAA amino acids in PKU and WT mice on control and LNAA supplemented diets are presented in S3 Table. In both PKU and WT mice, brain serine and glycine concentrations on LNAA supplemented diet were lower compared to control diets (*p*<0.05), although this did not reach statistical significance for glycine in PKU mice on LNAA supplementation compared to high-protein diet (*p* = 0.064). In addition, in WT mice only, brain lysine concentrations were lower (*p*<0.01), whereas brain taurine concentrations were higher on LNAA supplementation compared to control diets (*p*<0.05).

### Brain monoaminergic neurotransmitters

**Fig 5:**
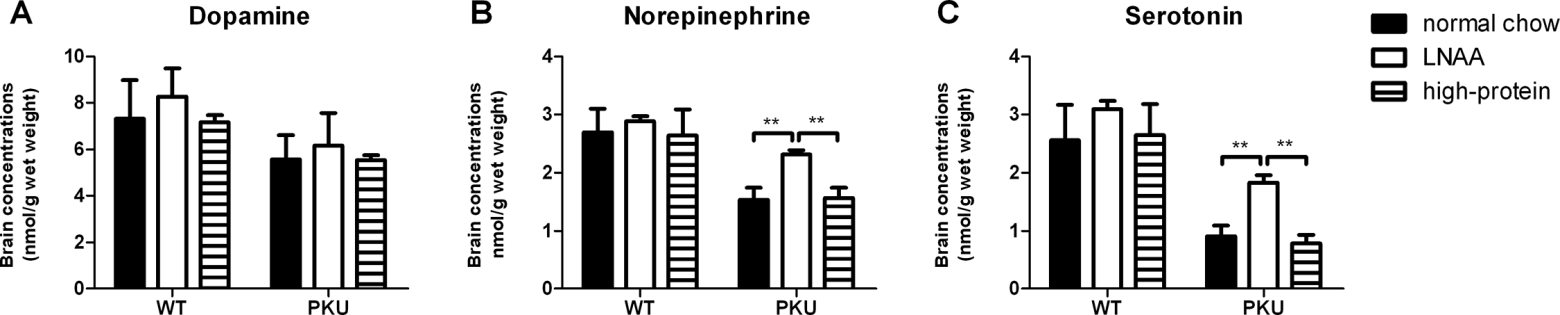
Brain monoaminergic neurotransmitter concentrations. Brain concentrations of A) dopamine, B) norepinephrine, C) serotonin in WT and PKU mice after six weeks of receiving different dietary treatments. Numbers of mice on normal chow, LNAA supplemented diet, and high-protein diet were n = 13, n = 12, and n = 14 for WT mice respectively, while being n = 15, n = 13, and n = 15 for PKU mice. Untransformed data are expressed as mean ± SEM. **p<0.01; §§ *p*<0.01 compared to WT mice on normal chow.

Brain monoaminergic neurotransmitter and associated metabolite concentrations in PKU and WT mice are depicted in Fig 5. In the catecholamine pathway, two-way ANOVA analyses showed significant main effects of genotype on brain dopamine and norepinephrine concentrations (*p*<0.01 for both). Brain dopamine and norepinephrine concentrations in PKU mice on normal chow were reduced to 76% and 57%, respectively, of concentrations in WT mice on the same diet. In the serotonergic pathway, significant main effects of genotype were observed on brain concentrations of serotonin and its associated metabolite 5-hydroxyindoleacetic acid (5-HIAA) (*p*<0.01 for both). Brain serotonin and 5-HIAA concentrations in PKU mice on normal chow were decreased to 35% and 26%, respectively, of concentrations in corresponding WT mice.

Dietary treatment had no significant effect on brain catecholamine or serotonin concentrations in WT mice, while in PKU mice it did. A significant main effect of dietary treatment was observed on brain catecholamine, serotonin, and 5-HIAA concentrations (*p*<0.01 for all but dopamine, *p*<0.05 for dopamine). Moreover, two-way ANOVA analyses showed a significant interaction between genotype and dietary treatment on brain norepinephrine, serotonin, and 5-HIAA (*p*<0.01 for all), but not dopamine concentration (*p* = 0.766).

In the catecholamine pathway, LNAA supplementation in PKU mice resulted in significantly higher brain norepinephrine concentrations compared to normal chow (*p*<0.01), and partially restored its deficit to an average of 86% of concentrations in WT mice on normal chow. In contrast, brain dopamine concentrations did not significantly differ in PKU mice between the dietary treatments (*p* = 0.307). In WT mice, no significant differences were observed between any of the dietary treatments for neither dopamine (*p* = 0.104) nor norepinephrine (*p* = 0.283).

In the serotonergic pathway, in PKU mice on LNAA supplementation, both brain serotonin and 5-HIAA concentrations were significantly increased when compared to control diets (*p*<0.01), partially restoring their concentrations to an average of 71% and 67%, respectively, of concentrations in WT mice on normal chow. In WT mice on LNAA supplementation, brain serotonin concentrations tended to be higher compared to normal chow (*p* = 0.051), and brain 5-HIAA concentrations tended to be higher compared to high-protein diet (*p* = 0.097), but both did not reach statistical significance.

### Relation between plasma and brain Phe

**Fig 6:**
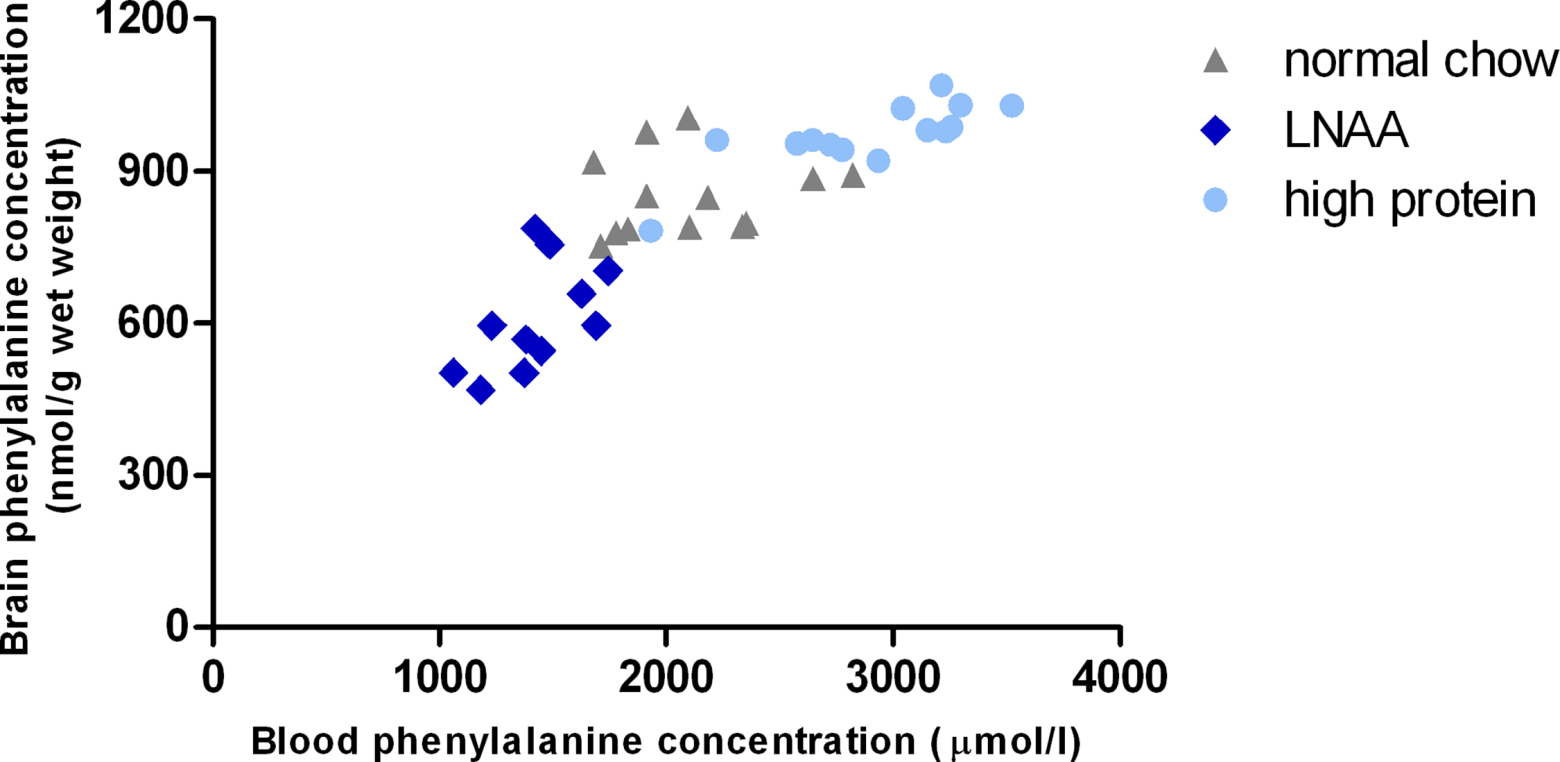
Plasma versus brain Phe concentrations in PKU mice on different dietary treatments. Relationship between plasma Phe and brain Phe concentrations in PKU mice on normal chow (n = 13), LNAA supplemented diet (n = 12), and high-protein diet (n = 14).

To investigate whether the reduction of brain Phe concentrations on LNAA supplementation in PKU mice was primarily related to an effect at the BBB or especially related to reduced blood Phe concentrations, the relationship between blood and brain Phe concentrations was assessed in PKU mice on LNAA supplementation and control diets (Fig 6). Multiple linear regression analysis showed that brain Phe concentrations in PKU mice were significantly predicted (adjusted R^2^ = 0.801, F = 75.525, *p*<0.01) by both blood Phe concentrations (B = 0.127, SE B = 0.026, *β* = 0.521) and LNAA supplemented diet (B = -166.940, SE B = 39.006, *β* = -0.451). Brain non-Phe LNAA concentrations in PKU mice on normal chow, LNAA supplementation, and high-protein diet did not show a clear relationship with either their respective blood concentrations nor with blood Phe concentrations (data not shown).

### Relation between brain monoaminergic neurotransmitters and their precursors

**Fig 7:**
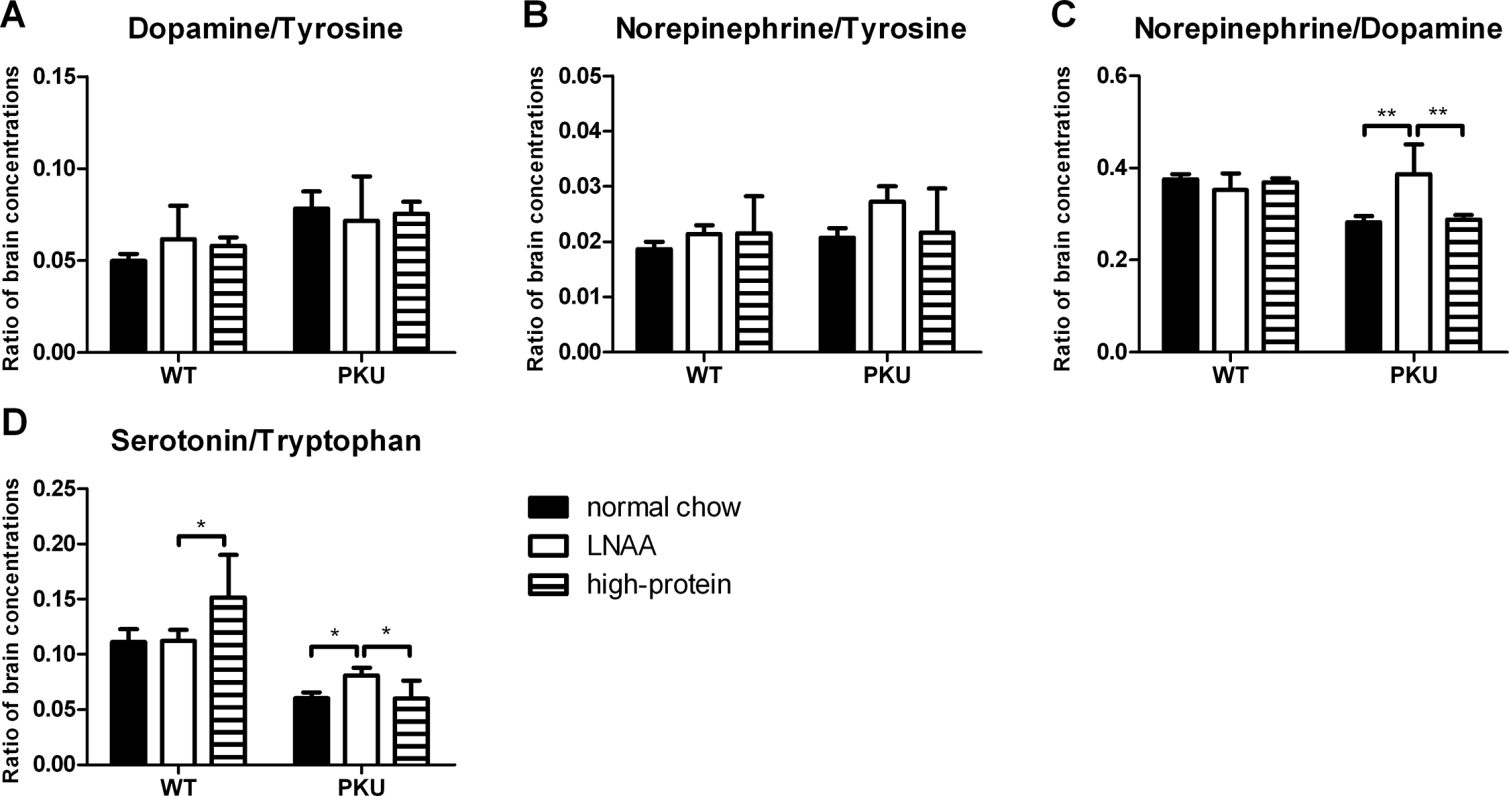
Ratios of brain monoaminergic neurotransmitters to precursors. Ratios of brain A) dopamine/tyrosine, B) norepinephrine/tyrosine, C) norepinephrine/dopamine, and D) serotonin/tryptophan concentrations in WT and PKU mice after six weeks of receiving different dietary treatments. Numbers of mice on normal chow, LNAA supplemented diet, and high-protein diet were n = 13, n = 12, and n = 14 for WT mice respectively, while being n = 14, n = 12, and n = 14 for PKU mice. Untransformed data are expressed as mean ± SEM. *p<0.05; **p<0.01; and § *p*<0.05; §§ *p*<0.01 compared to WT mice on normal chow.

To investigate whether the increase of brain serotonin and norepinephrine on LNAA supplementation in PKU mice was primarily due to (1) increased brain availability of their precursors, or to (2) enhanced conversion of their precursors, brain monoaminergic neurotransmitters were assessed in relation to their respective (amino acid) precursors (Fig 7).

Two-way ANOVA analyses showed a significant main effect of genotype on ratios of brain dopamine/tyrosine, norepinephrine/dopamine, and serotonin/tryptophan (*p*<0.01 for all), but not norepinephrine/tyrosine concentrations (*p* = 0.184). Ratios of brain dopamine/tyrosine were increased, while rations of brain norepinephrine/dopamine and serotonin/tryptophan concentrations were reduced in PKU compared to WT mice on normal chow.

A significant main effect of dietary treatment was observed on ratios of brain norepinephrine/dopamine concentrations only (*p*<0.01). In addition, two-way ANOVA analyses showed a significant interaction between genotype and dietary treatment on ratios of brain norepinephrine/dopamine and serotonin/tryptophan concentrations (*p*<0.01 for both). In PKU mice on LNAA supplementation, ratios of brain norepinephrine/dopamine were increased compared to control diets (*p*<0.01). Also, ratios of brain serotonin/tryptophan concentrations in PKU mice on LNAA supplementation were higher as compared to control diets (*p*<0.05).

## Discussion

This study is the first to investigate all hypothesized biochemical treatment effects of LNAA supplementation using one LNAA supplementation regimen and a single experimental design. Besides reducing blood Phe concentrations, the present study showed that LNAA supplementation without dietary Phe restriction in PKU mice could directly improve brain biochemistry through three mechanisms: 1) reducing brain Phe concentrations, 2) attenuating the brain deficiencies of some, but not all, LNAA, and 3) increasing brain serotonin and norepinephrine, but not dopamine concentrations. Before discussing these results in more detail, we will first address some methodological issues.

Firstly, the present experiment was performed in *Pah-enu2* (PKU) mice, a well-established model that resembles the genetics, biochemistry and neurobiology of classical PKU in humans. Biochemically, PKU mice show high blood and brain Phe concentrations in combination with brain non-Phe LNAA and monoaminergic neurotransmitter deficits that also characterize human PKU biochemistry. K_m_-values for LNAA transport at the BBB have not been determined for (PKU) mice [33]. However, K_m_-values for individual LNAA as determined *in vivo* in rats and in human brain capillaries showed a significant correlation [34], while being approximately 8–40 times lower for humans than for rats. This could imply that competition between Phe and non-Phe LNAA for transport across the BBB takes place at lower plasma concentrations in humans than in rats (and maybe mice), so that LNAA supplementation might be even more effective in PKU patients. One of the important advantages of this PKU mouse model over clinical studies is the possibility to measure not only Phe (that can be determined in humans by magnetic resonance spectroscopy (MRS)) but also non-Phe LNAA and monoaminergic neurotransmitter concentrations in brain (that at present cannot be measured by MRS).

Secondly, the LNAA supplemented diet used was based on the study by Pietz et al. (1999) that investigated the effect of concomitant LNAA supplementation during an oral Phe challenge on brain Phe uptake and EEG activity in PKU patients [26]. The LNAA supplementation regimen used by Pietz et al. consisting of equal amounts (150 mg/kg body weight) of all non-Phe LNAA except for threonine, in total, approximated the daily dietary protein intake for adults. To translate this acute regimen for PKU patients to chronic treatment in PKU mice, the total amount of added LNAA in the present study was equal to the amount of natural protein in the basal diet. In full accordance with the study by Pietz et al., threonine was not supplemented, even though Sanjurjo et al. (2003) have shown threonine supplementation alone (50 mg/kg/d) reduced blood Phe concentrations by 36% in PKU patients [35].

### LNAA supplementation reduces blood Phe concentrations

Although LNAA supplementation is suggested to improve brain metabolism primarily by restoring the unbalanced LNAA transport at the BBB, LNAA supplementation in PKU mice was also found to significantly reduce blood Phe concentrations to 67% of concentrations on normal chow. This is in accordance with previous studies on LNAA supplementation in PKU mice showing plasma Phe reductions to 47.0–63.5% of concentrations in untreated PKU controls [23,24]. LNAA supplementation has been hypothesized to exert this effect through competition with Phe for uptake at the gut-blood barrier [21], or through increased Phe utilization due to higher net protein synthesis [21]. In support of this last hypothesis, food intake of PKU mice during the final weeks of the experiment was significantly lower on LNAA supplemented diet than on control diets, while body weight did not significantly differ. This may suggest that LNAA supplementation indeed induced anabolism in PKU mice, thereby demanding a lower dietary protein (and thus food) intake, and by that contributing to the observed plasma Phe reduction. As expected, plasma concentrations of supplemented LNAA in PKU mice were all increased. Surprisingly, however, plasma tyrosine concentrations in PKU mice remained low-normal on the currently applied LNAA supplemented diet. Even when correcting for the 20% of the supplemented tyrosine that is assumed not to be absorbed in the gastrointestinal tract [21], tyrosine intake still was 3.5 times higher in the LNAA supplemented diet compared to normal chow. Similar to plasma tyrosine concentrations, brain tyrosine concentrations were not significantly increased on LNAA supplemented diet either, while brain norepinephrine—the end product of tyrosine-derived neurotransmitter metabolism—was significantly increased on LNAA supplemented diet. Therefore, a possible explanation might be that all supplemented tyrosine was used to partly restore the profound brain norepinephrine deficiency, and thereby did not result in increased plasma and brain tyrosine concentrations.

### LNAA supplementation reduces brain Phe concentrations, while attenuating brain deficiencies of some but not all non-Phe LNAA

In brain, Phe concentrations on LNAA supplementation in PKU mice were significantly reduced by 26% compared to concentrations on normal chow. This is in good agreement with previous studies on LNAA supplementation in both PKU patients [25] and mice [24], showing brain Phe reductions of 20–46%. Moreover, it may well support the finding of a clear competitive effect on Phe transport across the BBB in PKU patients by Pietz et al. (1999) using a comparable LNAA supplement [26]. As besides brain Phe, blood Phe concentrations were also reduced in PKU mice on LNAA supplementation, the question arises whether the reduced brain Phe concentrations might be due to the reduced plasma Phe concentrations rather than a direct effect at the BBB level. Multiple linear regression analysis suggests, however, that LNAA supplementation reduced brain Phe concentrations in PKU mice through a combined effect of both plasma Phe reduction and enhanced competition at the BBB.

This is the first time that brain non-Phe LNAA concentrations have been reported in response to LNAA supplementation in PKU. The LNAA supplementation regimen that was used, changed most brain non-Phe LNAA concentrations, restoring brain tryptophan in PKU mice to WT concentrations. Brain methionine concentrations were even significantly increased by 5.5-fold in PKU mice on LNAA supplementation compared to normal chow, corresponding with the similarly strong increases in blood. Although the cerebral and systemic effects and possible toxicity due to these strongly elevated methionine concentrations are not fully understood [36], at least these results warrant against indiscriminate supplementation of methionine in PKU. Also, brain histidine concentrations in PKU mice were even further increased on LNAA supplementation. From the fact that histidinaemia, in which brain histidine concentrations are much more increased, is not associated with any brain dysfunction, we may conclude that this elevation of brain histidine as observed in PKU mice probably does not have clinical significance [37]. On the other hand, brain concentrations of threonine, which was not included in the LNAA supplement, were significantly reduced in both PKU and WT mice on LNAA supplemented diet. This further supports the idea that highly unbalanced LNAA intake may induce brain deficiencies of some LNAA.

It can be concluded from these results that LNAA supplementation in PKU mice indeed attenuates brain Phe concentrations and attenuates brain deficiencies of (at least some) non-Phe LNAA. At the same time, results suggest that the relationships between brain non-Phe LNAA concentrations and their respective plasma concentrations as well as plasma Phe concentrations are complex and differ for each non-Phe LNAA, given the amount of LNAA supplementation used in this study. Development of the optimal LNAA supplementation regimen that can both effectively reduce brain Phe concentrations and improve brain concentration of all non-Phe LNAA therefore clearly deserves further research.

### LNAA treatment improves brain serotonin and norepinephrine, but not dopamine, concentrations

Besides its effect on brain LNAA concentrations, LNAA supplementation in PKU mice significantly increased brain serotonin from 35% to 71% of concentrations in WT mice. Also, brain norepinephrine in PKU mice on LNAA supplementation increased from 57% to 86% of concentrations in WT mice, whereas brain dopamine concentrations remained unchanged. Although brain monoaminergic neurotransmitter concentrations in response to LNAA supplementation have not been reported previously, a recent study on LNAA supplementation in PKU patients showed increased melatonin (a serotonin metabolite) concentrations in plasma and urine, which—according to the authors- could be a possible new marker for brain serotonin synthesis in PKU patients [29]. As C57Bl/6 is one of many mouse strains being deficient in melatonin [38], unfortunately, we were unable to correlate brain serotonin and plasma melatonin concentrations. Regarding the clinical importance of brain monoaminergic neurotransmitters in PKU, traditionally, especially brain dopamine deficiency has been associated with cognitive and mood disturbances in PKU [39,40]. However, brain norepinephrine impairments may have been underestimated, while cerebral norepinephrine abnormalities have been associated with many (neuro)psychiatric disorders [41].

Both insufficient precursor availability and impaired TH and TPH activity by inhibition of excessive brain Phe concentrations have been hypothesized to account for the brain monoaminergic neurotransmitter deficits in PKU. The present results suggest that the relative contribution of each of these mechanisms may be different for the dopaminergic and serotoninergic pathways in PKU. Regarding the brain catecholamine deficiencies, the increased ratio of brain dopamine/tyrosine and unaffected ratio of brain norepinephrine/tyrosine in PKU mice could be explained in two ways, each supporting one of the two aforementioned main theories on brain monoaminergic neurotransmitter deficiencies in PKU. Firstly, it may indicate that insufficient brain tyrosine availability rather than inhibition of TH by high Phe would be responsible for the brain catecholamine deficiencies observed in PKU. This would support the report by Fernstrom et al. (2007) concluding that increased brain Phe is not likely to impair catecholamine synthesis in PKU, whereas low brain tyrosine does [42]. In consequence, this would implicate that even higher blood tyrosine concentrations may be needed to restore brain dopamine concentrations. Secondly, the increased ratio of brain dopamine/tyrosine and unaffected ratio of brain norepinephrine/tyrosine in PKU compared to WT mice on normal chow may be explained by the fact that brain dopamine is not exclusively derived from brain tyrosine. This would support the report by Ikeda et al. (1967) showing that TH, at least in vitro, could synthesize catecholamines also from Phe, which is abundant in the PKU brain. This would suggest that insufficient precursor amino acid availability in brain would not be the primary mechanism underlying reduced dopamine concentrations, but inhibition of TH by high Phe is [43]. In consequence, this would imply that further reduction of brain Phe concentrations would probably be most effective to increase brain dopamine concentrations in PKU. Regarding the observed serotonin deficits in PKU, the reduced ratios of brain serotonin/tryptophan in PKU mice suggest that inhibition of TPH by high Phe does play an important role in the cerebral serotonin impairments characterizing PKU. This is in good agreement with the *in vitro* observation that Phe inhibits TPH more strongly than TH [44]. To further discriminate between the importance of both hypothesized mechanisms underlying brain monoaminergic neurotransmitter impairments in PKU, future studies need to investigate the effects of selective brain tyrosine and tryptophan increase and selective brain Phe reduction on monoaminergic neurotransmitter concentrations in PKU mice.

To conclude, this study was the first to investigate all hypothesized biochemical treatment objectives of LNAA supplementation in PKU. Results in PKU mice showed that LNAA supplementation improves brain biochemistry in PKU by three synergistic mechanisms. Thereby, this study provides proof-of-concept for LNAA supplementation as a possible alternative treatment strategy for PKU that improves brain biochemistry by targeting the unbalanced LNAA transport across the BBB. Before clinical application should be considered, however, further optimization of LNAA treatment with regard to the LNAA being supplemented and their dose is required, taking into account all three brain biochemical treatment objectives.

## Supporting Information

S1 ARRIVE Checklist ARRIVE guidelines checklist. (PDF)

S1 Table Average LNAA intakes of the different experimental groups (mg/g body weight/day). (DOC)

S2 Table Plasma non-LNAA amino acid concentrations after six weeks of receiving different diets. (DOC)

S3 Table Brain non-LNAA amino acid concentrations after six weeks of receiving different diets. (DOC)
