# Supplementary material for: Large Neutral Amino Acid Supplementation Exerts Its Effect through Three Synergistic Mechanisms: Proof of Principle in Phenylketonuria Mice
Source: PLoS One. 2015 Dec 1;10(12):e0143833. doi: 10.1371/journal.pone.0143833 (PMC4666635; doi:10.1371/journal.pone.0143833)
Supplement: S1 Table — (DOC) [file pone.0143833.s002.doc]

**Supplemental table 1. Average LNAA intakes of the different experimental groups (mg/g body weight/day)**

|  | WT | | | | | | | | | | |  | | PKU | | | | | | | | | |
| --- | --- | --- | --- | --- | --- | --- | --- | --- | --- | --- | --- | --- | --- | --- | --- | --- | --- | --- | --- | --- | --- | --- | --- |
|  | normal  chow | | | LNAA  diet | | | | high-protein  diet | | | |  | | normal  chow | | | | LNAA  diet | | | high-protein  diet | | |
| Phenylalanine | 0.92 | ± | 0.09 | | 0.88 | ± | 0.07 | | 1.81 | ± | 0.15 | |  | | 1.15 | ± | 0.18 | 0.97 | ± | 0.12 | 2.19 | ± | 0.13 |
| Tyrosine | 0.74 | ± | 0.08 | | 2.91 | ± | 0.24 | | 1.80 | ± | 0.15 | |  | | 0.92 | ± | 0.14 | 3.20 | ± | 0.38 | 2.17 | ± | 0.13 |
| Valine | 1.14 | ± | 0.12 | | 3.62 | ± | 0.30 | | 2.37 | ± | 0.19 | |  | | 1.42 | ± | 0.22 | 3.98 | ± | 0.47 | 2.85 | ± | 0.17 |
| Isoleucine | 0.91 | ± | 0.09 | | 3.32 | ± | 0.28 | | 1.84 | ± | 0.15 | |  | | 1.13 | ± | 0.18 | 3.65 | ± | 0.43 | 2.22 | ± | 0.13 |
| Leucine | 1.68 | ± | 0.17 | | 4.06 | ± | 0.34 | | 3.44 | ± | 0.28 | |  | | 2.09 | ± | 0.32 | 4.46 | ± | 0.53 | 4.14 | ± | 0.25 |
| Methionine | 0.46 | ± | 0.05 | | 2.85 | ± | 0.24 | | 1.03 | ± | 0.08 | |  | | 0.57 | ± | 0.09 | 3.14 | ± | 0.37 | 1.24 | ± | 0.07 |
| Histidine | 0.49 | ± | 0.05 | | 2.75 | ± | 0.23 | | 1.01 | ± | 0.08 | |  | | 0.61 | ± | 0.10 | 3.03 | ± | 0.36 | 1.22 | ± | 0.07 |
| Threonine | 0.82 | ± | 0.08 | | 0.83 | ± | 0.07 | | 1.68 | ± | 0.14 | |  | | 1.02 | ± | 0.16 | 0.91 | ± | 0.11 | 2.03 | ± | 0.12 |

LNAA intake is given in mg/g body weight/day, expressed as mean ± SD.

Dietary intake is not shown for Tryptophan, as this could not be measured in the food pellets.

Numbers of mice on normal chow, LNAA supplemented diet, and high-protein diet were n=13, n=13, and n=14 for WT mice respectively, while being n=15, n=14, and n=15 for PKU mice.
