# Supplementary material for: Large Neutral Amino Acid Supplementation Exerts Its Effect through Three Synergistic Mechanisms: Proof of Principle in Phenylketonuria Mice
Source: PLoS One. 2015 Dec 1;10(12):e0143833. doi: 10.1371/journal.pone.0143833 (PMC4666635; doi:10.1371/journal.pone.0143833)
Supplement: S2 Table — (DOC) [file pone.0143833.s003.doc]

**Supplemental table 2. Plasma non-LNAA amino acid concentrations after six weeks of receiving different diets**

|  | WT | | | | | | | | | | |  | | PKU | | | | | | | | | |
| --- | --- | --- | --- | --- | --- | --- | --- | --- | --- | --- | --- | --- | --- | --- | --- | --- | --- | --- | --- | --- | --- | --- | --- |
|  | Normal  chow | | | LNAA  diet | | | | High-protein  diet | | | |  | | Normal  chow | | | | LNAA  diet | | | High-protein  diet | | |
| Taurine | 425 | ± | 169 | | 513 | ± | 267 | | 334 | ± | 161 | |  | | 294 | ± | 120_a_ | 348 | ± | 72^#^ | 256 | ± | 66^#^ |
| Aspartic acid | 19 | ± | 7 | | 16 | ± | 7 | | 16 | ± | 8 | |  | | 17 | ± | 8 | 16 | ± | 9 | 12 | ± | 4 |
| Serine | 203 | ± | 46** | | 144 | ± | 36**^#^ | | 182 | ± | 39^#^ | |  | | 189 | ± | 54 | 172 | ± | 51 | 164 | ± | 46 |
| Asparagine | 71 | ± | 20 | | 61 | ± | 21 | | 60 | ± | 17 | |  | | 64 | ± | 31 | 68 | ± | 28 | 49 | ± | 19 |
| Glutamate | 48 | ± | 30 | | 41 | ± | 39 | | 36 | ± | 18 | |  | | 31 | ± | 16_a_ | 32 | ± | 19 | 26 | ± | 10 |
| Glutamine | 585 | ± | 81 | | 558 | ± | 75 | | 539 | ± | 108 | |  | | 550 | ± | 157* | 506 | ± | 86 | 422 | ± | 70* |
| Proline | 191 | ± | 95 | | 159 | ± | 76 | | 232 | ± | 89 | |  | | 153 | ± | 67 | 165 | ± | 51 | 169 | ± | 72 |
| Glycine | 249 | ± | 30** | | 130 | ± | 50**^##^ | | 222 | ± | 26^##^ | |  | | 232 | ± | 89** | 144 | ± | 52**^#^ | 196 | ± | 45^#^ |
| Alanine | 749 | ± | 209 | | 672 | ± | 259 | | 636 | ± | 194 | |  | | 587 | ± | 142_a_ | 695 | ± | 178^##^ | 482 | ± | 160^##^ |
| Citrulline | 77 | ± | 14 | | 86 | ± | 19 | | 76 | ± | 17 | |  | | 92 | ± | 17_a_** | 88 | ± | 15 | 77 | ± | 16* |
| Ornithine | 67 | ± | 19 | | 55 | ± | 15 | | 76 | ± | 30 | |  | | 72 | ± | 33 | 69 | ± | 26 | 61 | ± | 16 |
| Lysine | 550 | ± | 160** | | 300 | ± | 64**^##^ | | 552 | ± | 140^##^ | |  | | 426 | ± | 130_a_ | 322 | ± | 72 | 387 | ± | 97 |
| Arginine | 113 | ± | 29 | | 87 | ± | 17 | | 105 | ± | 30 | |  | | 77 | ± | 24_aa_ | 68 | ± | 20 | 81 | ± | 19 |

Plasma concentrations are expressed in µmol/l (mean ± SD).

Concentrations in PKU mice on normal chow are compared to WT mice on normal chow (_a_ <0.05 and _aa_<0.01).

Within the groups of WT and PKU mice, concentrations that differ between dietary treatment groups are indicated (*<0.05;

**<0.01; ^#^<0.05; and  ^##^<0.01).
