# Supplementary material for: Large Neutral Amino Acid Supplementation Exerts Its Effect through Three Synergistic Mechanisms: Proof of Principle in Phenylketonuria Mice
Source: PLoS One. 2015 Dec 1;10(12):e0143833. doi: 10.1371/journal.pone.0143833 (PMC4666635; doi:10.1371/journal.pone.0143833)
Supplement: S3 Table — (DOC) [file pone.0143833.s004.doc]

**Supplemental table 3. Brain non-LNAA amino acid concentrations after six weeks of receiving different diets**

|  | WT | | | | | | | | | | |  | | PKU | | | | | | | | | |
| --- | --- | --- | --- | --- | --- | --- | --- | --- | --- | --- | --- | --- | --- | --- | --- | --- | --- | --- | --- | --- | --- | --- | --- |
|  | Normal  chow | | | LNAA  diet | | | | High-protein  diet | | | |  | | Normal  chow | | | | LNAA  diet | | | High-protein  diet | | |
| Taurine | 9913 | ± | 702* | | 10610 | ± | 488*^##^ | | 9839 | ± | 534^##^ | |  | | 10324 | ± | 1054 | 11041 | ± | 831 | 10933 | ± | 684 |
| Aspartic acid | 4833 | ± | 402 | | 5036 | ± | 450 | | 4936 | ± | 433 | |  | | 4471 | ± | 459_a_ | 4501 | ± | 348 | 4556 | ± | 317 |
| Serine | 1012 | ± | 96** | | 617 | ± | 102**^##^ | | 1012 | ± | 93^##^ | |  | | 1137 | ± | 98_aa_** | 872 | ± | 174**^##^ | 1121 | ± | 146^##^ |
| Asparagine | 80 | ± | 9 | | 72 | ± | 12 | | 78 | ± | 14 | |  | | 76 | ± | 23 | 72 | ± | 14 | 74 | ± | 8 |
| Glutamate | 10334 | ± | 562 | | 10157 | ± | 553 | | 10336 | ± | 439 | |  | | 9624 | ± | 636_aa_ | 9627 | ± | 825 | 9554 | ± | 679 |
| Glutamine | 4702 | ± | 1885 | | 3821 | ± | 677^#^ | | 4846 | ± | 992^#^ | |  | | 4264 | ± | 1862 | 4405 | ± | 1293 | 3663 | ± | 743 |
| Proline | 131 | ± | 55 | | 151 | ± | 55 | | 131 | ± | 65 | |  | | 146 | ± | 88 | 138 | ± | 76 | 144 | ± | 70 |
| Glycine | 1496 | ± | 152** | | 1153 | ± | 143**^##^ | | 1467 | ± | 157^##^ | |  | | 1817 | ± | 373_aa_* | 1466 | ± | 285* | 1724 | ± | 265 |
| Alanine | 874 | ± | 108 | | 840 | ± | 120 | | 816 | ± | 130 | |  | | 760 | ± | 84_aa_ | 745 | ± | 123 | 776 | ± | 95 |
| GABA | 3876 | ± | 490 | | 3941 | ± | 514 | | 3834 | ± | 473 | |  | | 3584 | ± | 514 | 3596 | ± | 503 | 3716 | ± | 525 |
| Lysine | 328 | ± | 37** | | 282 | ± | 28**^##^ | | 329 | ± | 42^##^ | |  | | 304 | ± | 48 | 289 | ± | 49 | 304 | ± | 40 |
| Arginine | 260 | ± | 69 | | 265 | ± | 65 | | 261 | ± | 80 | |  | | 225 | ± | 86 | 237 | ± | 90 | 232 | ± | 73 |

Brain concentrations are expressed in nmol/g wet weight (mean ± SD).

Concentrations in PKU mice on normal chow are compared to WT mice on normal chow (_a_ <0.05 and _aa_<0.01).

Within the groups of WT and PKU mice, concentrations that differ between dietary treatment groups are indicated (*<0.05;

**<0.01; ^#^<0.05; and  ^##^<0.01).
